# Erythropoietin mediates re-programming of endotoxin-tolerant macrophages through PI3K/AKT signaling and protects mice against secondary infection

**DOI:** 10.3389/fimmu.2022.938944

**Published:** 2022-08-09

**Authors:** Xue Zhang, Dan He, Jialin Jia, Feihong Liang, Jie Mei, Wenhua Li, Tingting Liu, Zhiyu Wang, Yu Liu, Fengxue Zhang, Zhiren Zhang, Bangwei Luo

**Affiliations:** ^1^ Research Center of Integrative Medicine, School of Basic Medical Sciences, Guangzhou University of Chinese Medicine, Guangzhou, China; ^2^ Medical College, Chongqing University, Chongqing, China; ^3^ Department of Medical Science, Shunde Polytechnic, Foshan, China; ^4^ Institute of Immunology, Army Medical University, Chongqing, China

**Keywords:** endotoxin tolerance, sepsis, macrophages, HIF-1α, erythropoietin

## Abstract

Initial lipopolysaccharide (LPS) exposure leads to a hypo-responsive state by macrophages to a secondary stimulation of LPS, known as endotoxin tolerance. However, recent findings show that functions of endotoxin-tolerant macrophages are not completely suppressed, whereas they undergo a functional re-programming process with upregulation of a panel of molecules leading to enhanced protective functions including antimicrobial and tissue-remodeling activities. However, the underlying molecular mechanisms are still elusive. Erythropoietin (EPO), a glycoprotein regulated by hypoxia-inducible factor 1α (HIF-1α), exerts anti-inflammatory and tissue-protective activities. Nevertheless, the potential effects of EPO on functional re-programming of endotoxin-tolerant macrophages have not been investigated yet. Here, we found that initial LPS exposure led to upregulation of HIF-1α/EPO in macrophages and that EPO enhanced tolerance in tolerized macrophages and mice as demonstrated by suppressed proinflammatory genes such as *Il1b*, *Il6*, and *Tnfa* after secondary LPS stimulation. Moreover, we showed that EPO improved host protective genes in endotoxin-tolerant macrophages and mice, such as the anti-bacterial genes coding for cathelicidin-related antimicrobial peptide (*Cnlp*) and macrophage receptor with collagenous structure (*Marco*), and the tissue-repairing gene vascular endothelial growth factor C (*Vegfc*). Therefore, our findings indicate that EPO mediates the functional re-programming of endotoxin-tolerant macrophages. Mechanistically, we found that PI3K/AKT signaling contributed to EPO-mediated re-programming through upregulation of *Irak3* and *Wdr5* expression. Specifically, IL-1 receptor-associated kinase 3 (IRAK3) was responsible for inhibiting proinflammatory genes *Il1b*, *Il6*, and *Tnfa* in tolerized macrophages after LPS rechallenge, whereas WDR5 contributed to the upregulation of host beneficial genes including *Cnlp*, *Marco*, and *Vegfc*. In a septic model of mice, EPO pretreatment significantly promoted endotoxin-tolerant re-programming, alleviated lung injury, enhanced bacterial clearance, and decreased mortality in LPS-tolerized mice after secondary infection of *Escherichia coli*. Collectively, our results reveal a novel role for EPO in mediating functional re-programming of endotoxin-tolerant macrophages; thus, targeting EPO appears to be a new therapeutic option in sepsis and other inflammatory disorders.

## Introduction

Sepsis is a life-threatening pathology that arises from dysregulated host inflammatory response to severe bacterial infection, trauma, or cancer. The annual prevalence of sepsis worldwide is estimated at 19 million and it is a leading cause of death in intensive care units globally (1). Although great advance of therapeutic strategy has been made during the past 20 years, severe sepsis-related mortality still remains high at 20%–30% approximately (2). Therefore, novel effective therapeutic strategy is urgently required to improve the outcomes of septic patients. Severe proinflammatory response in sepsis results in multiple organ dysfunction in clinical patients; however, a number of clinical trials designed for blocking proinflammatory cytokines have failed in septic patients (3, 4). Nevertheless, increasing studies have found that patients who survived the initial acute stage of sepsis often developed an immuno-suppressive state, resulting in increased risks of detrimental secondary infections which are held responsible for high mortality in sepsis (5). These facts indicate that anti-inflammatory therapy alone might not be sufficient for the successful treatment of sepsis and it will be of great interest to achieve attenuation of cytokine response while enhancing protective immune function against secondary infections.

Endotoxin tolerance refers to a refractory state of macrophages induced by initial exposure to lipopolysaccharide (LPS) in response to a secondary dose of LPS (6). The endotoxin-tolerant macrophages are characterized by suppressed secretion of proinflammatory cytokines such as TNF-α, IL-6, and IL-1β after a secondary LPS stimulation (7). However, in animal models of experimental sepsis induced by bacterial challenge, for example, *Salmonella typhimurium*, *Pseudomonas aeruginosa*, and *Staphylococcus aureus*, results from prior works demonstrated that, although the inflammatory response was suppressed by LPS pretreatment, LPS-tolerant mice showed enhanced bacterial clearance and improved survival, suggesting that LPS tolerance upregulated the immune function to clear pathogenic bacteria despite cytokine response being attenuated (8–10). In addition, accumulating *in vitro* studies show that, in LPS-tolerant human peripheral blood mononuclear cells and murine macrophages, although genes encoding proinflammatory mediators such as *Il1b*, *Il6*, and *Tnfa* are silenced, genes encoding antimicrobial effectors such as cathelicidin-related antimicrobial peptide (CRAMP) coding gene *Cnlp* and macrophage receptor with collagenous structure coding gene *Marco* remain inducible (11). Most recently, investigations in clinical patients with sepsis reveal that rather than globally suppressed, septic blood monocytes undergo a functional re-programming from proinflammatory to an endotoxin-tolerant state. This re-programming process includes not only suppression of proinflammatory cytokines but also upregulation of several important host protective genes, for example, the antimicrobial gene coding for hepcidin antimicrobial peptide (HAMP) and the tissue-remodeling gene vascular endothelial growth factor (VEGF) (12). Therefore, endotoxin tolerance is different from post-injury immunosuppression of clinical patients and it may serve as an important mechanism to suppress proinflammatory response while enhancing the innate immune clearance of pathogens and tissue-repairing functions. Thus, exploring the re-programming mechanism of endotoxin tolerance and the development of novel tolerance regulators will be of great significance to achieve better outcomes in treating patients with sepsis and other diseases.

Hypoxia-inducible factor 1α (HIF-1α), a key transcriptional factor in the regulation of hypoxic response, plays a critical role in the development of immune re-programming in sepsis. For instance, recent studies reveal that expression of HIF-1α is significantly upregulated in monocytes isolated from sepsis patients and that HIF-1α mediates functional re-programming of monocytes by enhancing protective functions like phagocytosis, antimicrobial activity, and tissue-remodeling functions (12). However, the specific underlying molecular mechanisms have not been entirely clarified. Activation of HIF-1α is known to regulate numerous hypoxia-sensitive genes, for example, erythropoietin (EPO), a hematopoietic hormone that acts by increasing oxygen availability *via* binding to its receptor EPOR (13). Recently, expression of EPOR has been found in several non-hematopoietic systems, especially the immune system such as macrophages (14). Treatment of EPO has been shown to exhibit potent anti-inflammatory functions in LPS-activated proinflammatory macrophages by inhibition of NF-κB (15). Moreover, EPO has been shown to enhance phagocytotic activity of macrophages (16). In addition, expression of EPOR has also been found in other non-hematopoietic systems such as the central nervous system, and EPO has been shown great tissue-protective effects in neurons (17, 18). However, there is still no report on the potential effect of EPO in the regulation of endotoxin tolerance. In this regard, we ask whether EPO is involved in the functional re-programming of endotoxin-tolerant macrophages and explore the underlying mechanisms.

## Materials

Salidroside (SAL) (CAS 10338-51-9, purity >98%) and 5-aza-2′-deoxycytidine (5-AZA) were purchased from Solarbio (Beijing, China). LPS (*Escherichia coli* 055:B5) was purchased from Sigma Chemical Co. (St. Louis, MO, USA). Dulbecco’s modified Eagle’s medium (DMEM) and fetal bovine serum (FBS) were obtained from Invitrogen-Gibco (Grand Island, NY, USA). The neutralizing antibody to EPO (anti-EPO-16, Clone 16F1H11) was purchased from Stemcell Technologies (Vancouver, Canada). Recombinant mouse macrophage colony-stimulating factor (M-CSF) was purchased from Sinobiological (Beijing, China). Recombinant human EPO (rhEPO) was purchased from Sunshine Pharmaceutical (Shenyang, China). BAY87-2243 and MK2206 were purchased from Beyotime Biotechnology (Shanghai, China). WDR5-0103 was purchased from Selleck (Houston, TX, USA). *Irak3* siRNA(m) was purchased from Santa Cruz Biotechnology (Dallas, TX, USA).

### Animals and mouse models

Wild-type C57BL/6 male mice approximately 8–12 weeks old were purchased from Army University Experimental Animal Center and acclimatized for 1 week before use. Epor^loxp/loxp^LysM-Cre^+/+^ mice were referred to as *EPOR-cKO* mice described previously (19). All mice were housed and bred in the animal facility at the Army Medical University under specific pathogen–free conditions. Rodent laboratory chow and tap water were provided and maintained under controlled conditions with a temperature of 24°C ± 1°C and a 12-h light/12-h dark cycle. All of the procedures were in strict accordance with the guide. *E. coli* serotype O6:K2:H1 cultured in Luria–Bertani (LB) broth was harvested at mid-log phase (OD_600_ ≈ 0.8; 5 × 10^9^ CFU/ml) and then washed twice in sterile PBS. Sepsis was induced *via* an intraperitoneal injection of 10^7^ indicated CFU of *E. coli* into the abdominal cavity of mice. The peritoneal lavage fluids were collected aseptically by irrigating the peritoneal cavity with sterile PBS. Bacterial loads in peritoneal cavity were assessed to evaluate the bacterial clearance using the method described previously (19).

### Cell culture

The RAW 264.7 murine macrophage cell line was obtained from the China Cell Line Bank (Beijing, China). Bone marrow cells were obtained from the femur and tibia of mice aged 8–12 weeks. After erythrocytes lysis and centrifugation, they were cultured in DMEM medium containing M-CSF (50 ng/ml) for 3 days. On day 4, fresh DMEM medium containing M-CSF into cell culture was added. After being cultured in DMEM medium containing M-CSF for a total of 6 days, adherent cells as bone marrow–derived macrophages (BMDMs) were collected. All cells were cultured in DMEM supplemented with 10% heat-inactivated FBS at 37°C under a humidified atmosphere of 5% CO2.

### Real-time quantitative PCR

RNA was isolated from cultured cells or mice tissue samples with the RNA fast 200 Kit (Fastagen, Shanghai, China) according to the manufacturer’s instructions. Reverse transcription was performed using a reverse transcription kit (Takara, Tokyo, Japan). qRT-PCR was performed using SYBR Green qPCR Master Mix (MedChemExpress, Monmouth Junction, NJ, USA). qRT-PCR was run on the CFX96 detection system (Bio-Rad Laboratories, Hemel Hempstead, UK); gene expression for each sample was normalized to β-actin for the mouse reference gene, and the differences were determined using the 2^-ΔΔCT^ calculation. The sequences of primers used are listed in **Supplementary methods**.

### Histological assessment

Lung samples were harvested, fixed in 4% paraformaldehyde, dehydrated, bisected, mounted in paraffin, and sectioned for H&E staining according to the manufacturer’s protocol (Sigma-Aldrich, St Louis, MO, USA). For histological evaluation, the lung injury scores were quantified as previously described (19).

### Statistics

All values in the figures and text are expressed as means ± SEM. Significance was calculated using one-way or two-way ANOVA with Tukey’s *post-hoc* test for multiple comparisons or Student’s *t*-test for two groups meeting the normal distribution criteria. Survival rate was analyzed *via* the log-rank test. For all statistical analyses, the statistical significance was represented by a single asterisk (P < 0. 05), two asterisks (P < 0. 01), three asterisks (P < 0. 001), or four asterisks (P < 0. 0001) using GraphPad Prism 9.0.

## Results

### Blockade of endogenous EPO impaired re-programming of endotoxin-tolerant macrophages

**Figure 1:**
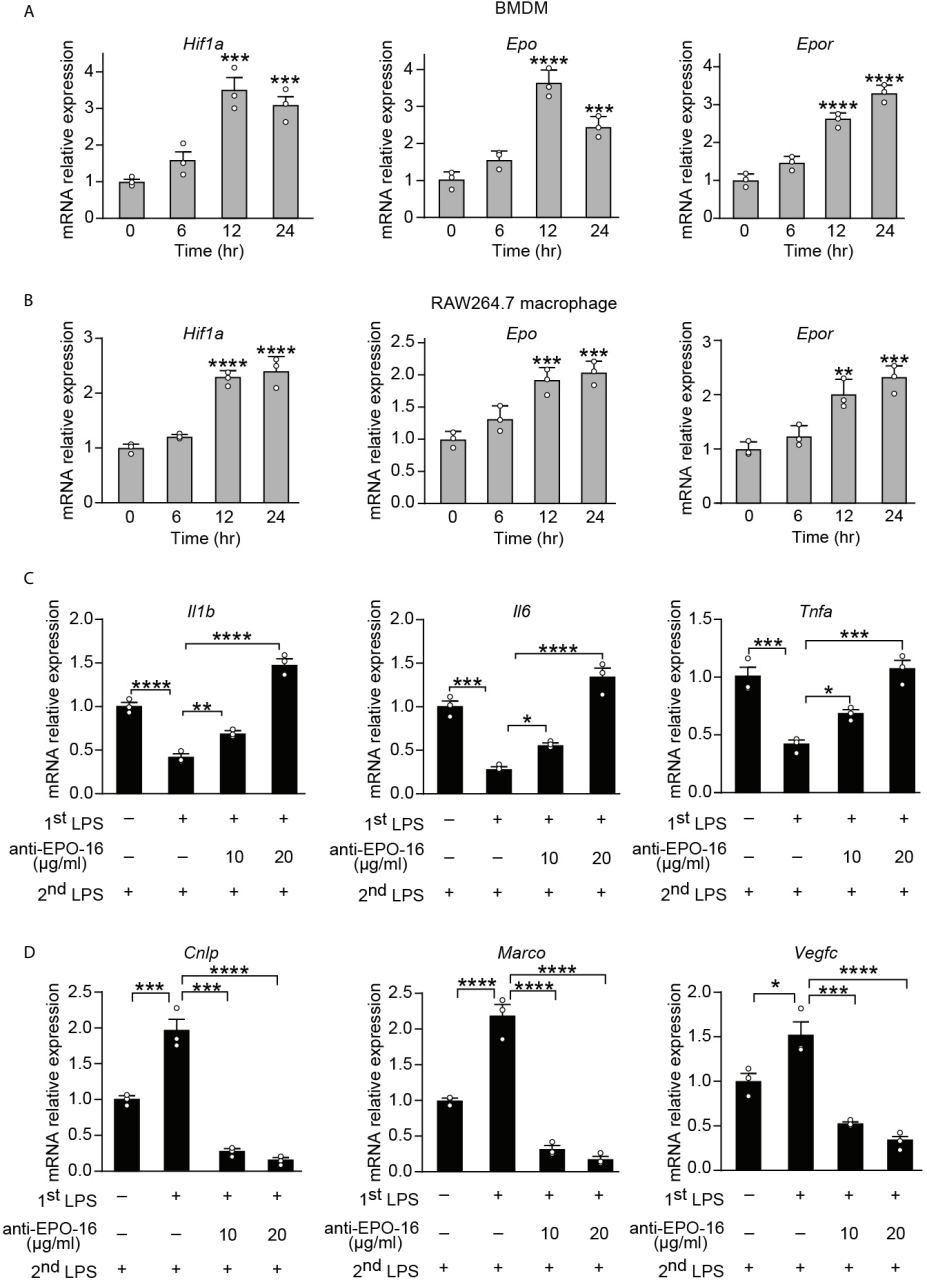
Blockade of endogenous EPO impaired re-programming of endotoxin-tolerant macrophages. **(A, B)**: *In vitro* cultured BMDMs from WT C57BL/6 mice **(A)** or mouse RAW264.7 macrophages **(B)** were incubated with LPS (100 ng/ml) for 0, 6, 12, and 24 h, and mRNA levels of *Hif1a*, *Epo*, and *Epor* in macrophages were measured by qRT-PCR (n = 3). (**C**, **D)**: *In vitro* cultured RAW264.7 macrophages were pretreated with PBS (non-tolerant), LPS (100 ng/ml) (tolerant), or LPS (100 ng/ml) + anti-EPO-16 (10 or 20 μg/ml) for 24 h. Then, cells were washed with PBS twice. Subsequently, cells were given with a secondary LPS stimulation (10 ng/ml) for 6 h, and the mRNA levels of *Il1b*, *Il6*, *Tnfa* **(C)**, *Cnlp*, *Marco*, and *Vegfc* **(D)** were measured by qRT-PCR (n = 3). Data are representative of three independent experiments. Results were expressed as means ± SEM. *P < 0.05, **P < 0.01, ***P < 0.001, and ****P < 0.0001 (one-way ANOVA with Tukey’s *post-hoc* test for multiple comparisons).

Tolerance by macrophages to endotoxin can be elicited *in vitro* by long-term exposure to LPS. In order to determine whether the expression of endogenous EPO is induced by initial LPS exposure during the establishment of endotoxin tolerance, purified BMDMs from healthy wild-type (WT) C57BL/6 mice and a mouse cell line RAW264.7 macrophages were incubated with LPS (100 ng/ml) for 0, 6, 12, and 24 h, and mRNA levels of *Hif1a*, *Epo*, and its receptor *Epor* were measured by real-time quantitative reverse transcription PCR (qRT-PCR). Consistent with the previous study which established that HIF-1α expression was significantly upregulated in blood monocytes from septic patients (12), our results showed that gene expression of *Hif1a* was significantly elicited in BMDMs and RAW264.7 macrophages at 12 and 24 h by LPS tolerization (**Figures 1A, B**). We next measured the mRNA expression of *Ep*o and its receptor *Epor* in LPS-tolerized macrophages. As demonstrated in **Figures 1A, B**, *Ep*o and *Epor* mRNAs at 12 and 24 h were markedly upregulated in LPS-tolerized BMDMs and RAW264.7 macrophages. Therefore, our data demonstrated that the macrophage EPO pathway was induced endogenously by initial LPS exposure during the establishment of endotoxin tolerance. We thus hypothesized that the endogenously induced EPO might play a role in regulating the functional re-programming of endotoxin-tolerant macrophages. To address this question, a neutralizing antibody to EPO (anti-EPO-16) was used in our *in vitro* experiments to block endogenous EPO activities. Mice RAW264.7 macrophages were exposed to primary dose of LPS (100 ng/ml) for 24 h to induce tolerance, together with anti-EPO-16 or isotype IgG. Then, cells were washed with PBS twice and, subsequently, cells were given with a secondary LPS stimulation (10 ng/ml) for 6 h. As demonstrated in **Figure 1C**, our results showed that exposure of RAW264.7 macrophages to initial LPS induced an endotoxin-tolerant state indicated by the significant suppression of *Il1b*, *Il6*, and *Tnfa* mRNA after secondary LPS stimulation. However, anti-EPO-16 at a concentration of 10 μg/ml or higher significantly reversed the suppressed mRNA expression of *Il1b*, *Il6*, and *Tnfa* in tolerized macrophages following LPS rechallenge. Another hallmark of endotoxin-tolerant re-programming is the upregulation of antimicrobial and tissue-repairing genes. Our results showed that after LPS restimulation, tolerized macrophages showed a notable alteration in the pattern of host beneficial genes, with a remarkable upregulation of anti-bacterial genes, for example, *Cnlp* and *Marco*, as well as tissue-repairing gene *Vegfc* compared to non-tolerant macrophages (**Figure 1D**). However, when endogenous EPO was neutralized with anti-EPO-16, LPS-tolerant macrophages failed to upregulate expression of *Cnlp*, *Marco*, and *Vegfc* in response to secondary LPS stimulation (**Figure 1D**). Therefore, our data demonstrated that blockade of endogenous EPO significantly dampened the formation of functional re-programming in tolerized macrophages.

### EPO-mediated functional re-programming of endotoxin-tolerant macrophages

**Figure 2:**
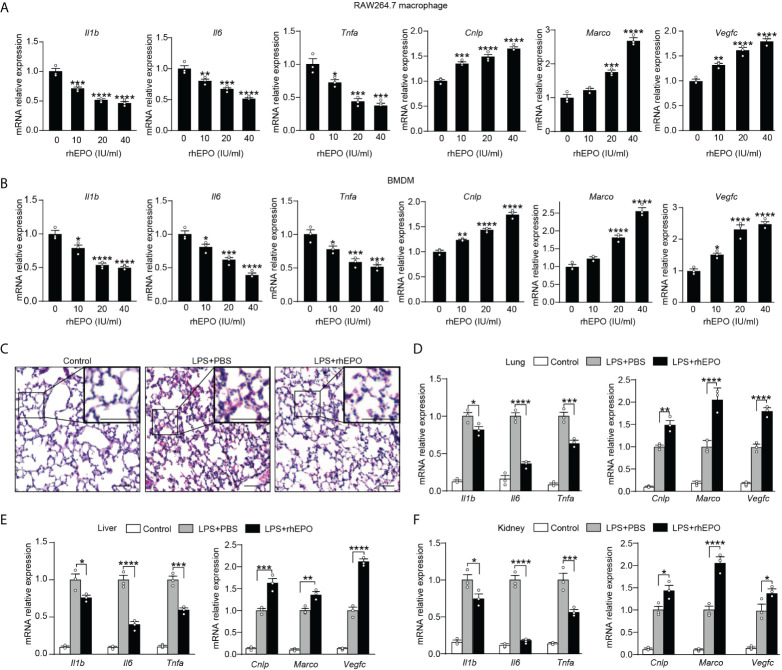
Exogenous EPO promoted endotoxin-tolerant re-programming.**(A, B)**: *In vitro* cultured RAW264.7 macrophages **(A)** or BMDMs from WT C57BL/6 mice **(B)** were incubated with different dose of rhEPO (0, 10, 20, and 40 IU/ml) in the presence of LPS (100 ng/ml) for 24 h, and then cells were washed with PBS twice followed by a secondary LPS stimulation (10 ng/ml) for 6 h; gene expression was measured by qRT-PCR (n = 3). **(C**–**F)**: WT C57BL/6 mice were intraperitoneally injected with LPS (1 mg/kg) together with rhEPO (5,000 IU/kg) or PBS for 24 h, and then mice were intraperitoneally given with a secondary LPS injection (10 mg/kg) for 6 h. Control mice were injected with PBS only. **(C)**: Lung specimens stained with H&E (bar = 100 μm, n = 3). **(D**–**F)**: qRT-PCR assay of gene expression in mice’s lung **(D)**, liver **(E)**, and kidney **(F)** (n = 3). Data are representative of three independent experiments. Results were expressed as means ± SEM. *P < 0.05, **P < 0.01, ***P < 0.001, and ****P < 0.0001. Statistics: one-way ANOVA **(A, B)** or two-way ANOVA **(D-F)** with Tukey’s *post-hoc* test for multiple comparisons.

Having established that endogenous EPO played an important role in the development of functional re-programming of endotoxin-tolerant macrophages, we anticipated that exogenous EPO could re-program LPS-tolerized macrophages in response to secondary LPS stimulation. To test directly whether exogenous EPO affects macrophage re-programming, we treated macrophages with rhEPO during the LPS-tolerization period. Specifically, BMDMs or RAW264.7 macrophages were exposed to a primary dose of LPS (100 ng/ml) for 24 h to induce tolerance, together with different doses of rhEPO or PBS. Then, cells were washed with PBS twice and, subsequently, cells were given with a secondary LPS stimulation (10 ng/ml) for 6 h. As shown in **Figure 2A, B** and **Figure S1A**, pretreatment of rhEPO during LPS-tolerization dose-dependently decreased mRNA expression and protein secretion of proinflammatory cytokines IL-1β, IL-6, and TNF-α but upregulated mRNA levels of protective genes *Cnlp*, *Marco*, and *Vegfc* in LPS-restimulated macrophages. Thus, our results showed that incubation of exogenous EPO during initial LPS tolerization promoted macrophage re-programming by suppressing inflammatory response while increasing host protective genes in response to secondary LPS stimulation. EPO is known to act *via* its receptor EPOR, we thus further verified the role of EPO-mediated endotoxin-tolerant re-programming with BMDMs from *EPOR-cKO* mice, a myeloid-specific *EPOR* knockout mice (19). As shown in **Figure S1B**, pretreatment of rhEPO failed to elicit endotoxin tolerance in tolerized BMDMs from *EPOR-cKO* mice, verifying that EPO mediated re-programming of endotoxin-tolerant macrophages *via* its receptor EPOR. Collectively, our *in vitro* studies demonstrated that exogenous EPO enhanced endotoxin-tolerant re-programming of tolerized macrophages in an EPOR-dependent manner.

*In vivo* effects of exogenous EPO on endotoxin tolerance were examined in WT C57BL/6 mice and *EPOR-cKO* mice. Mice were injected with LPS (1 mg/kg, i.p.) to induce tolerance, together with rhEPO (5,000 IU/kg) or an equal volume of PBS for 24 h, and then they were given with a secondary LPS injection (10 mg/kg, i.p.) for 6 h. The lung is one of the most vulnerable organs injured by sepsis, we examined the lung specimens after secondary LPS challenge by staining with H&E. As shown in **Figure 2C** and **Figure S1C**, lung tissues from the EPO group showed less interstitial edema, coagulation, and inflammatory cell infiltration, indicating a less inflammatory response and an increased tissue-protective effect by EPO pretreatment. Overexpression of proinflammatory cytokines contributes to lung injury in septic mice, and we observed a significantly lower level of proinflammatory *Il1b*, *Il6*, and *Tnfa* mRNA in the lungs from rhEPO-pretreated mice (**Figure 2D**), whereas mRNA expression of host beneficial genes *Cnlp*, *Marco*, and *Vegfc* was significantly increased in rhEPO-pretreated group (**Figure 2D**). Similar results were observed in the liver, kidney, and spleen of rhEPO-pretreated mice (**Figures 2E, F**, **Figure S1D**). Additionally, the *in vivo* roles of exogenous EPO were further validated in *EPOR-cKO* mice, and we found that pretreatment of rhEPO failed to induce endotoxin tolerance in tolerized *EPOR-cKO* mice to secondary LPS challenge (**Figures S1E–H**). Thus, our animal experiment results indicated that exogenous EPO mediated re-programming of endotoxin tolerance *in vivo* and this effect was macrophage EPOR–dependent.

### EPO re-programmed endotoxin-tolerant macrophages through PI3K/AKT pathway *via* upregulation of IRAK3 and WDR5

**Figure 3:**
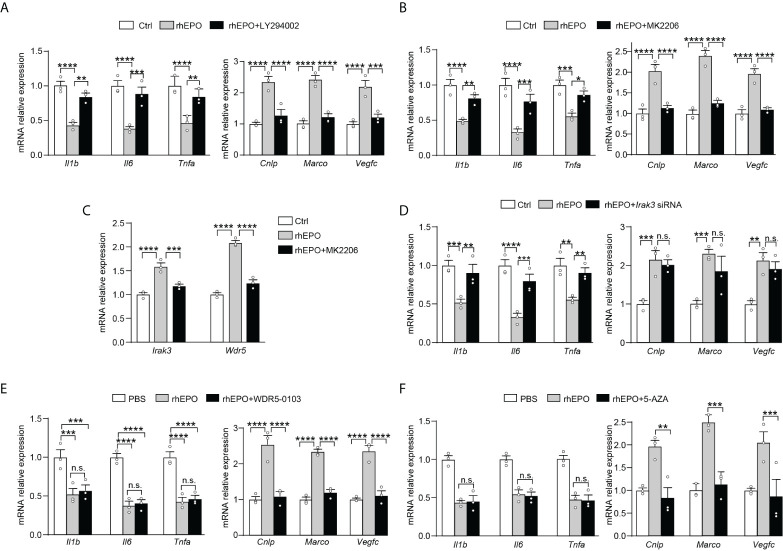
EPO mediated re-programming of endotoxin-tolerant macrophages through PI3K/AKT pathway *via* upregulation of IRAK3 and WDR5 **(A, B)**: *In vitro* cultured RAW264.7 macrophages were incubated with PBS, rhEPO (40 IU/ml), and rhEPO (40 IU/ml) + LY294002 (20 μM) **(A)** or PBS, rhEPO (40 IU/ml), and rhEPO (40 IU/ml) + MK2206 (5 μM) **(B)** the in presence of LPS (100 ng/ml) for 24 h, and then cells were washed with PBS twice followed by a secondary LPS stimulation (10 ng/ml) for 6 h; gene expression was measured by qRT-PCR (n = 3). **(C)**: *In vitro* cultured RAW264.7 macrophages were incubated with PBS, rhEPO (40 IU/ml), or rhEPO (40 IU/ml) + MK2206 (5 μM) in the presence of LPS (100 ng/ml) for 24 h, and then gene expression of *Irak3* and *Wdr5* was measured by qRT-PCR (n = 3). **(D**–**F)**: RAW264.7 macrophages were incubated with PBS, rhEPO (40 IU/ml), and rhEPO (40 IU/ml) + *Irak3* siRNA (50 nM) **(D)**; PBS, rhEPO (40 IU/ml), and rhEPO (40 IU/ml) + WDR5-0103 (500 nM) **(E)**; or PBS, rhEPO (40 IU/ml), and rhEPO (40 IU/ml) + 5-AZA (5 μM) **(F)** in the presence of LPS (100 ng/ml) for 24 h, and then cells were washed with PBS twice followed by a secondary LPS stimulation (10 ng/ml) for 6 h; gene expression was measured by qRT-PCR (n = 3). Data are representative of three independent experiments. Results were expressed as means ± SEM. *P < 0.05, **P < 0.01, ***P < 0.001, and ****P < 0.0001. n.s., not statistically significant. Statistics: two-way ANOVA with Tukey’s *post-hoc* test for multiple comparisons. "n.s." stands for "not statistically significant“.

We next explored pathways that participated in EPO-mediated re-programming of endotoxin-tolerant macrophages. One of the well-known EPO signaling pathways is the phosphatidyl-inositol-3 kinase (PI3K) and its downstream target protein kinase B (AKT) pathway (20), which is discovered to negatively regulate LPS signaling in macrophages (21). Therefore, in the present study, we sought to determine whether PI3K/AKT pathway was responsible for the EPO-mediated re-programming of endotoxin-tolerant macrophages. To clarify this question, the PI3K inhibitor LY294002 and AKT inhibitor MK2206 were examined in our experiments. As shown in **Figures 3A, B**, inhibition of PI3K/AKT pathway with LY294002 (20 μM) or MK2206 (5 μM) remarkably reversed the suppressed expression of proinflammatory *Il1b*, *Il6* and *Tnfa*, whereas significantly impaired the upregulated expression of host beneficial genes *Cnlp*, *Marco* and *Vegfc* by rhEPO in tolerized RAW264.7 macrophages after secondary LPS stimulation. Thus, our results indicated that PI3K/AKT pathway was indeed responsible for the EPO-mediated re-programming of endotoxin-tolerant macrophage. Negative regulators of LPS response, for instance, IL-1 receptor–associated kinase 3 (IRAK3), play important roles in endotoxin tolerance by limiting overexpression of proinflammatory cytokines (22). On the other hand, the upregulation of tissue-protective genes in endotoxin tolerance is associated with epigenetic mechanisms, for example, trimethylation of histone H3 methylated at K4 (H3K4me3) (11). Therefore, we asked whether these negative feedback regulators and epigenetic modulation enzymes might play critical roles in EPO-mediated endotoxin-tolerant re-programming. We treated LPS-tolerized RAW264.7 macrophages with rhEPO during initial the LPS-tolerization period for 24 h; as shown in **Figure S2**, rhEPO significantly increased gene expression of *Irak3* and increased mRNA expression of WD repeat-containing protein 5 (*Wdr5*) which is essential for H3K4me3-specific histone methyl transferase activity. Additionally, this induction effect of EPO was inhibited by AKT inhibitor MK2206 (**Figure 3C**). Thus, our findings suggest that *Irak3* and *Wdr5* might be closely linked to the regulation of EPO-mediated macrophage re-programming. We then determined to investigate the role of IRAK3 and WDR5 in modulation of EPO-mediated re-programming in endotoxin-tolerant macrophages. As shown in **Figure 3D**, treatment with *Irak3* siRNA reversed the expression of proinflammatory *Il1b*, *Il6*, and *Tnfa* suppressed by rhEPO pretreatment in tolerized RAW264.7 macrophages triggered by LPS restimulation, whereas levels of host beneficial genes *Cnlp*, *Marco* and *Vegfc* were not affected. On the other hand, treatment of WDR5-0103, an inhibitor of WDR5, did not change the expression of proinflammatory *Il1b*, *Il6*, and *Tnfa* but specifically reduced levels of host protective genes *Cnlp*, *Marco*, and *Vegfc* (**Figure 3E**). Therefore, our results indicated that IRAK3 was responsible for the regulation of proinflammatory genes, whereas WDR5 was countable for modulating host protective genes in EPO-mediated re-programming of endotoxin-tolerant macrophages.

Previous experiments showed that treatment with a demethylating agent 5-AZA significantly decreased H3K4me3 in tolerized macrophages and reduced expression of *Marco* upon LPS rechallenge without change in the inflammatory component of immune tolerance (23). Thus, from the current data and that published by others, we speculated that this mechanism might also work for EPO-mediated expression of *Cnlp*, *Marco*, and *Vegfc* in tolerized macrophages. As shown in **Figure 3F**, we testified that in LPS-tolerized RAW264.7 macrophages, treatment with 5-AZA indeed significantly impaired EPO-mediated *Cnlp*, *Marco*, and *Vegfc* mRNA expression upon secondary LPS stimulation but did not affect the inflammatory response as measured by the expression of *Il1b*, *Il6*, and *Tnfa*. In aggregate, our results suggested that WDR5-induced methylation played a critical role in EPO-mediated functional re-programming of tolerized macrophages.

### Induction of endogenous EPO by salidroside promoted functional re-programming of endotoxin tolerance

**Figure 4:**
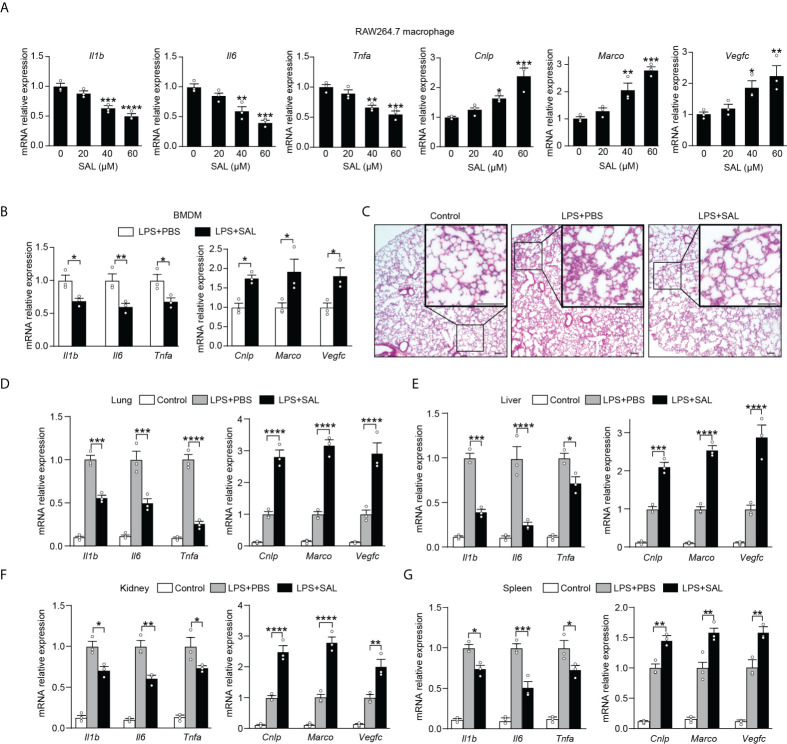
Induction of endogenous EPO by SAL improved endotoxin-tolerant re-programming **(A)**: *In vitro* cultured RAW264.7 macrophages were pretreated by LPS (100 ng/ml) together with different doses of SAL (0, 20, 40 and 60 μM) for 24 h. Then, cells were washed with PBS twice followed by a secondary LPS stimulation (10 ng/ml) for 6 h, and mRNA levels were measured by qRT-PCR (n = 3). **(B)**: *In vitro* cultured BMDMs from WT C57BL/6 mice were pretreated by LPS (100 ng/ml), together with SAL (60 μM) or PBS for 24 h. Then, cells were washed with PBS twice followed by a secondary LPS stimulation (10 ng/ml) for 6 h, and gene expression was measured by qRT-PCR (n = 3). For **(C**–**G)**: WT C57BL/6 mice were intraperitoneally injected with LPS (1 mg/kg) together with SAL (40 mg/kg) or PBS for 24 h, and then these mice were intraperitoneally given with a secondary LPS injection (10 mg/kg) for 6 h. Control mice were injected with PBS only. **(C)**: Lung specimens stained with H&E (bar = 100 μm, n = 3). **(D**–**G)**: qRT-PCR assay of gene expression in mice’s lung **(D)**, liver **(E)**, kidney **(F)**, and spleen **(G)** (n = 3). Results were expressed as means ± SEM. *P < 0.05, **P < 0.01, ***P < 0.001, and ****P < 0.0001. Statistics: one-way ANOVA **(A)** or two-way ANOVA **(D**–**G)** with Tukey’s *post-hoc* test for multiple comparisons.

While our findings promise beneficial effects of EPO, fear has also arisen that treatment of exogenous EPO may accelerate tumor growth in cancer patients in clinical studies (24, 25). In addition, there is an increased risk of thrombosis following EPO treatment and thus the pro-thrombotic effects of EPO therapy could not be neglected (26). To this end, it is of great interest to identify an alternative therapy without the potential risk of stimulating tumor growth or promoting thrombosis. SAL, a main bioactive component extracted from the root of *R. rosea* L., has long been used to prevent high-altitude sickness in traditional Chinese medicine (27). Recent studies have discovered that SAL is able to upregulate expression of HIF-1a and EPO even under non-hypoxia stress (28–31). Previous studies showed potent anti-inflammatory, anti-tumor, and anti-thrombosis activities of SAL (32–35). These findings, coupled with our data describing that blocking endogenous EPO impaired macrophage re-programming (**Figures 1C, D**), promoted us to conceive that SAL might serve as an optimal alternative therapy acting by inducing endogenous EPO without the risk of stimulating tumor growth or promoting thrombosis. In this event, we next determined to evaluate whether SAL was able to regulate the re-programming of endotoxin tolerance through induction of endogenous EPO in tolerized macrophages. To accomplish this, we treated RAW264.7 macrophages with different doses of SAL during LPS tolerization (100 ng/ml) for 24 h. As shown in **Figure S3A**, SAL incubation dose-dependently elicited gene expression of *Hif1a*, *Epo*, and *Epor* in LPS-tolerized RAW264.7 macrophages. Moreover, we conducted this experiment with BMDMs from healthy WT C57BL/6 mice and similar results were observed (**Figure S3B**), demonstrating that endogenous EPO could be induced by SAL in LPS-tolerized macrophages. Therefore, the pharmacological effects of SAL on mediating functional re-programming in endotoxin-tolerant macrophages were tested. As predicted, pretreatment of SAL dose-dependently reduced the expression of proinflammatory *Il1b*, *Il6*, and *Tnfa* mRNA but upregulated *Cnlp*, *Marco*, and *Vegfc* mRNA in tolerized RAW264.7 macrophages to LPS restimulation (**Figure 4A**). In addition, treatment with BAY87-2243 (20 μM), a potent HIF-1α inhibitor, eliminated the effects of SAL pretreatment on the re-programming of endotoxin-tolerant RAW264.7 macrophages (**Figure S3C**). Next, we performed *in vitro* experiments with BMDMs from healthy WT C57BL/6 mice and similar results were obtained (**Figures 4B** and **S3D**). To further testify whether SAL mediates the functional re-programming *via* endogenous EPO pathway, BMDMs from *EPOR*-cKO mice were used in our experiments and we found that after LPS tolerization and rechallenge, pretreatment of SAL failed to promote endotoxin tolerance in *EPOR*-cKO BMDMs (**Figure S3E**). Taken together, these data indicated that induction of endogenous EPO promoted the re-programming of endotoxin-tolerant macrophages *in vitro*.

We then investigated whether SAL could promote functional re-programming of endotoxin tolerance *in vivo* through the endogenous EPO pathway. Healthy WT C57BL/6 mice were injected with LPS (1 mg/kg, i.p.) to induce tolerance, together with SAL (40 mg/kg, i.p.) or an equal volume of PBS for 24 h, then these mice were given with a secondary LPS injection (10 mg/kg, i.p.) for 6 h. As shown in **Figures 4C** and **S4A**, the lung tissues from the SAL group showed less interstitial edema, coagulation, and inflammatory cell infiltration. As shown in **Figure 4D**, we found that SAL pretreatment lowered mRNA levels of proinflammatory *Il1b*, *Il6*, and *Tnfa*, whereas upregulated host beneficial genes *Cnlp*, *Marco*, and *Vegfc* in the lung. Similarly, we found lower levels of *Il1b*, *Il6*, and *Tnfa* mRNA and higher levels of *Cnlp*, *Marco*, and *Vegfc* mRNA in the liver, kidney, and spleen of SAL pretreated mice (**Figure 4E–G**). In addition, treatment with HIF-1α inhibitor BAY87-2243 (9 mg/kg, i.p.) markedly impaired SAL-induced endotoxin tolerance (**Figures S4B–E**). In accordance with *in vitro* results, SAL pretreatment failed to promote endotoxin tolerance in LPS-tolerized *EPOR-cKO* mice (**Figures S4F–I**), indicating that the *in vivo* effects of SAL in improving endotoxin tolerance were through macrophage EPOR. Taken together, our results provided a strong premise for SAL in mediated functional re-programming of endotoxin tolerance through the endogenous EPO pathway.

### EPO and SAL protected tolerized mice from secondary infection of *Escherichia coli* sepsis

**Figure 5:**
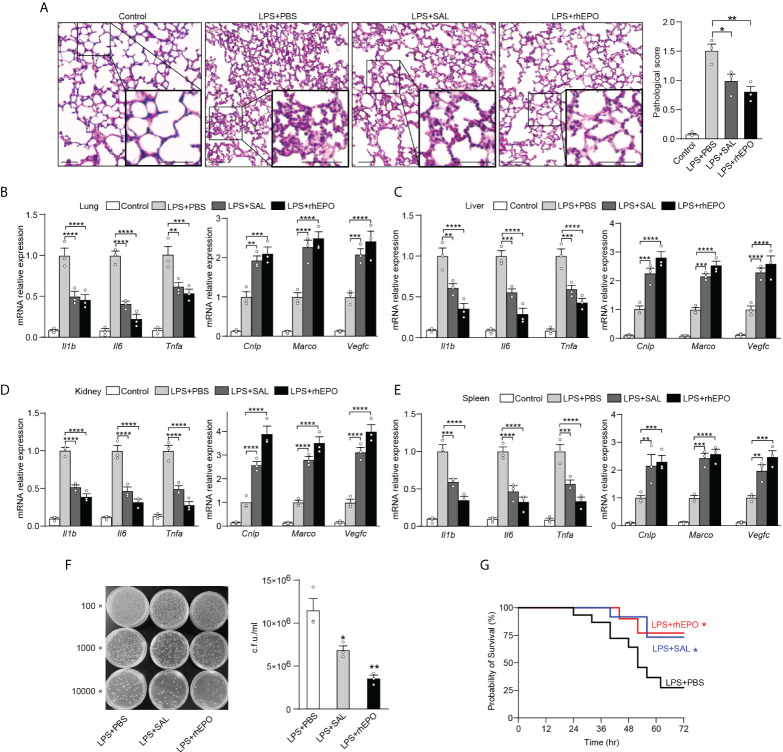
EPO and SAL protected LPS-tolerized mice from secondary infection of *E*. *coli* sepsis. WT C57BL/6 mice were intraperitoneally injected with LPS (1 mg/kg) + PBS, LPS (1 mg/kg) + rhEPO (5,000 IU/kg), or LPS (1 mg/kg) + SAL (40 mg/kg) for 24 h, and then mice were rechallenged with *E. coli* (10^7^ CFU, i.p.) for 6 h. Control mice were injected with PBS only. **(A)**: Lung specimens stained with H&E for histological evaluation (bar = 100 μm, n = 3). **(B–E)**: qRT-PCR evaluation of gene expression in mice’s lung **(B)**, liver **(C)**, kidney **(D)**, and spleen **(E)** (n = 3). **(F)**: Bacterial titers in peritoneal lavage fluids (n = 3). WT C57BL/6 mice were intraperitoneally injected with LPS (1 mg/kg) + PBS, LPS (1 mg/kg) + rhEPO (5,000 IU/kg), or LPS (1 mg/kg) + SAL (40 mg/kg) for 24 h, and then mice were rechallenged with *E. coli* (10^7^ CFU, i.p.) for 72 h for survival observation. **(G)**: Survival of mice (n = 15, log-rank test). Data are representative of three independent experiments. Results were expressed as means ± SEM. *P < 0.05, **P < 0.01, ***P < 0.001, and ****P < 0.0001. Statistics: one-way ANOVA **(A, F)** or two-way ANOVA **(B–E)** with Tukey’s *post-hoc* test for multiple comparisons.

Sepsis is often caused by a secondary bacterial infection in clinical settings. The incidence of gram-negative bacterial sepsis has risen significantly during the last decade. *E. coli* is one of the most frequent gram-negative bacterial pathogens of bloodstream infections and a major cause of death due to sepsis (36). Therefore, compared to LPS restimulation which is a commonly used method to establish an immune tolerance mice model, rechallenge with *E. coli* is a more clinically relevant experimental sepsis model to validate our findings in secondary infections. Thus, given the striking impact of EPO and SAL on LPS tolerance, we further examined the effects of EPO and SAL on the re-programming of immune tolerance in *E. coli–*induced septic mice. WT C57BL/6 mice were intraperitoneally injected with LPS (1 mg/kg) to induce tolerance, together with rhEPO (5,000 IU/kg), SAL (40 mg/kg), or PBS for 24 h, and then mice were challenged with secondary infection of *E. coli* (10^7^ CFU, i.p.) for 6 h followed by detection. As shown in **Figure 5A**, compared to the PBS group, lung tissues from the rhEPO or SAL group showed less pulmonary hemorrhage, infiltration of inflammatory cells, and degeneration in the lung tissue. As shown in **Figure 5B**, in mice lung tissues, mRNA levels of proinflammatory *Il1b*, *Il6*, and *Tnfa* in the rhEPO and SAL groups were significantly lower compared to the PBS group, whereas mRNA levels of *Cnlp*, *Marco*, and *Vegfc* in the rhEPO and SAL groups were increased. Similar results were obtained in the liver, kidney, and spleen samples of septic mice (**Figures 5C–E**). Thus, these data indicated that EPO and SAL mediated endotoxin-tolerant re-programming in LPS-tolerized mice after secondary infection of *E. coli*. In addition, we discovered that treatment of rhEPO or SAL promoted the re-programming of host immunity to an antimicrobial state (**Figure 5F**) as indicated by diminished bacterial loads in peritoneal exudates. Moreover, as shown in **Figure 5G**, LPS-tolerant mice subjected to secondary *E. coli* sepsis had a 75% mortality rate, and we found a higher survival rate by rhEPO and SAL pretreatment in LPS-tolerized mice after secondary infection of *E. coli*. Collectively, we found that EPO and SAL protected LPS-tolerized mice against secondary infection of *E. coli*–induced sepsis.

## Discussion

**Figure 6:**
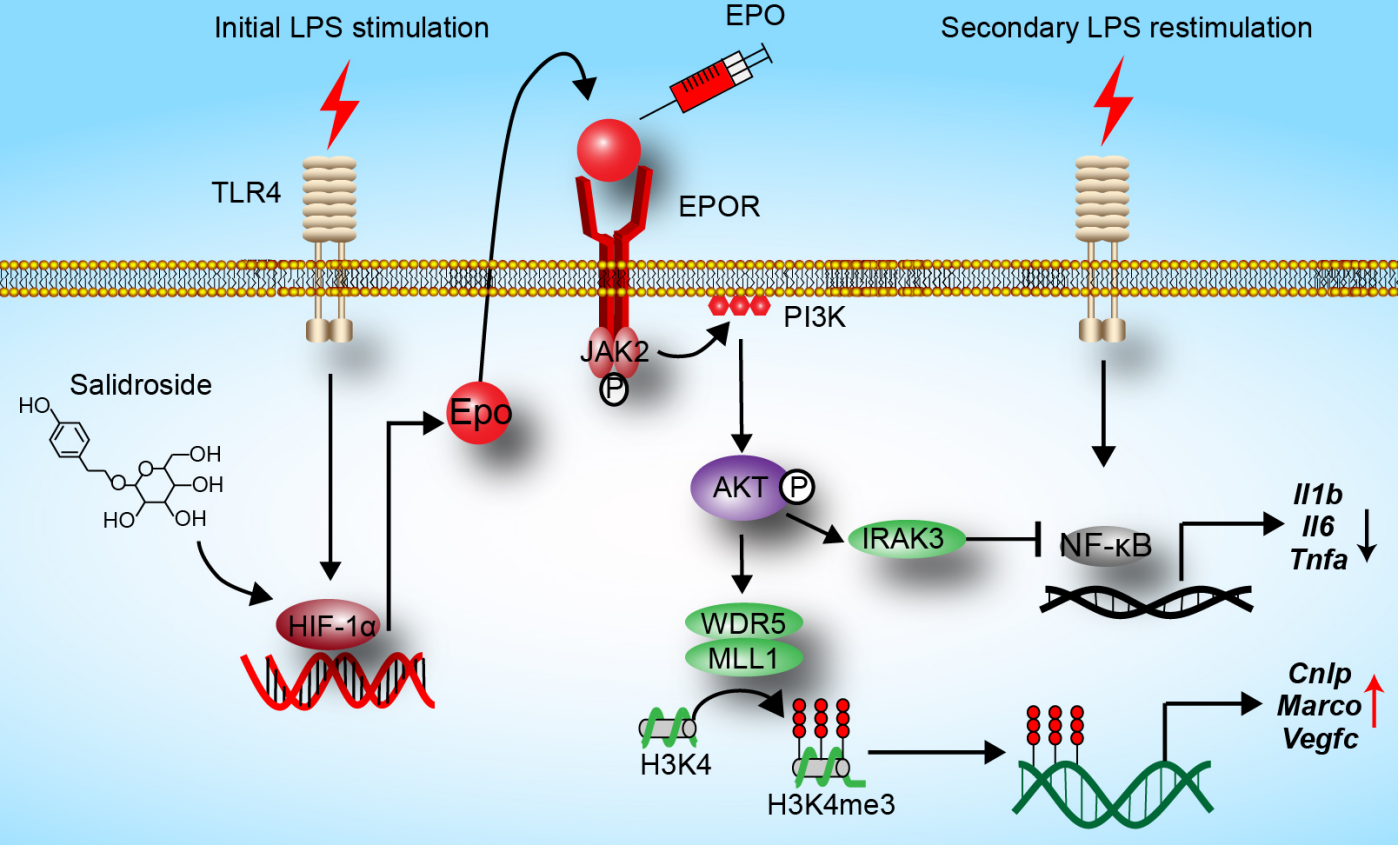
Hypothesis schema depicting the mechanism of endotoxin-tolerant re-programming mediated by EPO in macrophages. EPO is induced by initial LPS exposure through upregulation of HIF-1α. Binding of EPO to its receptor EPOR expressed on macrophages leads to activation of PI3K/AKT pathway, which further upregulated expressions of *Irak3* (a negative regulator of LPS response) and *Wdr5* (a core scaffolding component of histone methyltransferase complexes). When tolerized macrophages were challenged with a secondary dose of LPS, expression of proinflammatory genes such as *Il1b*, *Il6*, and *Tnfa* was robustly suppressed by IRAK3 *via* negative regulation of NF-κB, whereas the expression of host protective genes including *Cnlp*, *Marco*, and *Vegfc* in macrophages was upregulated by WDR5-induced histone methylation. In addition, SAL promotes re-programming of endotoxin-tolerant macrophages by inducing endogenous EPO through upregulation of *Hif1a*.

In the current study, we reveal that EPO is endogenously induced by initial LPS exposure in tolerized macrophages, and we show for the first time that EPO is a regulator of functional re-programming of endotoxin-tolerant macrophages (summarized in **Figure 6**). Endotoxin-tolerant macrophages are re-programmed by EPO to express less proinflammatory genes, for example, *Il1b*, *Il6*, and *Tnfa*, and more host protective genes such as *Cnlp*, *Marco*, and *Vegfc* in tolerized macrophages upon the secondary challenge of LPS. We established a mouse sepsis model by i.p. injection of *E. coli* to LPS-tolerized mice, and our results indicate that pretreatment of EPO mediated endotoxin-tolerant re-programming and protected mice from secondary infection of *E. coli*. Thus, EPO may be a potential target for the treatment of patients with sepsis.

Consistent with our findings in this study that EPO leads to a decreased level of proinflammatory cytokines in tolerized macrophages after LPS rechallenge, the anti-inflammatory activity of EPO has been reported in numerous models (37). On the other hand, we reveal a novel role of EPO in the functional re-programming of endotoxin-tolerant macrophages by upregulation of host protective genes including *Marco*, *Cnlp*, and *Vegfc*. Previous studies showed that the gene expression of *Marco* was selectively upregulated in LPS-tolerant macrophages and that MARCO contributed to the increased phagocytosis of tolerant macrophages (23, 38). *Cnlp* is responsible for the production of anti-bacterial effector CRAMP and an upregulated expression of *Cnlp* was also found in LPS-rechallenged macrophages (11, 39). In accordance with these studies, our data showed an upregulation of *Marco* and *Cnlp* gene expression by EPO and we observed an enhanced ability in bacterial clearance by EPO pretreatment in LPS-tolerized mice subjected to secondary infection of *E. coli*. Therefore, the upregulation of *Marco* and *Cnlp* by EPO-mediated re-programming of endotoxin-tolerant macrophages would be particularly helpful for septic patients who are at high risk of secondary infections.

VEGF plays a crucial role in wound healing and tissue repairing through the formation of blood and lymphatic vessels. VEGF-A regulates angiogenesis, whereas VEGF-C is responsible for lymphangiogenesis (40). Data from previous studies showed that the expression of *Vegfa* is upregulated in endotoxin-tolerant human monocytes (12). However, the enhanced circulating concentration of VEGF-A has been linked with sepsis severity and mortality (41). In addition, anti–VEGF-A antibody has been found to attenuate inflammation and decrease mortality in an experimental model of severe sepsis (42). Therefore, upregulation of VEGF-A seems not a protective mechanism in sepsis. In this regard, we focused on *Vegfc* rather than *Vegfa* in our current study. The gene expression of *Vegfc* was previously found to be upregulated in tolerized macrophages after LPS rechallenge (11). Moreover, VEGF-C secreted by macrophages was essential during tissue repair through lymphatic vessel formation (43, 44). Aside from targeting lymphatic vessels, recently, it has been reported that VEGF-C signaling in macrophages represents a self-control mechanism during anti-bacterial innate immunity and that VEGF-C protects mice against septic shock (45). These findings suggest that the upregulation of *Vegfc* might be an endotoxin tolerance–induced endogenous protective mechanism in sepsis. In our study, we found a dramatic increase of *Vegfc* by EPO in tolerized macrophages and mice following LPS restimulation. Therefore, the upregulated *Vegfc* by EPO-mediated re-programming in endotoxin-tolerant macrophages would be beneficial for the treatment of septic patients.

Identifying EPO-related pathways involved in re-programming may lead to potential targets for sepsis treatment. Therefore, our next focus was to identify the molecular mechanisms by which EPO mediated re-programming of endotoxin-tolerant macrophages. In previous studies, we found that EPO promoted infection resolution and ameliorated inflammatory response through a ligand-activated transcriptional factor peroxisome proliferator–activated receptor gamma (PPAR-γ) in macrophages (19). Nevertheless, recent study showed that PPAR-γ is not necessary for the development of LPS tolerance in macrophages (46). Therefore, we determined to examine other signaling pathways which may play important roles in the re-programming of endotoxin tolerance mediated by EPO. Binding of EPO triggers its receptor EPOR and activates multiple downstream signaling pathways including STAT5, MAPK, and PI3K/AKT (47). In the present study, we found that inhibition of PI3K/AKT with PI3K inhibitor LY294002 or AKT inhibitor MK2206 greatly dampened EPO-mediated functional re-programming of LPS-tolerant macrophages. In accordance with our observations, there have been increasing studies indicating that PI3K/AKT pathway is essential for LPS-induced tolerance. For example, it has been reported that blockade of PI3K with its inhibitor wortmannin reversed *in vivo* tolerance in LPS-pretreated mice (48, 49). For another example, results from Pik3r1^−/−^-deficient mice (PI3K activity reduced) and PTEN^−/−^ mice (AKT activity enhanced) demonstrated that PI3K/AKT pathway negatively regulated LPS signaling in macrophages and endotoxemic mice (50). Moreover, Androulidaki and colleagues reported that AKT^−/−^ macrophages exhibited increased responsiveness to LPS and that AKT^−/−^ mice did not develop endotoxin tolerance (51). However, previous experiments mainly focused on proinflammatory genes regulated by PI3K/AKT pathway in tolerized macrophages to secondary stimulation, and we revealed a novel role for PI3K/AKT signaling in EPO-mediated functional re-programming based on the significant enhancement of host protective genes including *Cnlp*, *Marco*, and *Vegfc* in endotoxin-tolerant macrophages.

Negative regulators play important roles in the development of endotoxin tolerance, for example, IRAK3 has been reported to suppress various Toll-like receptor (TLR)–mediated signal transduction in macrophages and essential for endotoxin tolerance (22, 52). In the present study, EPO markedly increased the expression of *Irak3* in LPS-tolerized macrophages and it was suppressed by AKT inhibitor MK2206. We next suppressed gene expression of *Irak3* with specific siRNA, and we found that this method effectively inhibited endotoxin tolerance induced by EPO, as reflected by the levels of *Il1b*, *Il6*, and *Tnfa* in tolerized RAW264.7 cells after LPS rechallenge. Consistent with our results, studies demonstrated that macrophages deficient in IRAK3 produced elevated levels of proinflammatory cytokines such as *Tnfa*, *Il*6, and *Il12* upon LPS challenge (22). However, we found that *Irak3* silencing did not change the expression of *Cnlp*, *Marco*, and *Vegfc* by EPO in LPS-restimulated macrophages. These observations demonstrated that other mechanisms could be responsible for the upregulation of protective genes in EPO-mediated re-programming of endotoxin-tolerant macrophages.

LPS-induced tolerance is an example of epigenetic re-programming and increasing studies have shown that chromatin modification plays a pivotal role in the modulation of re-programmed gene expression pattern. For example, H3K4me3, a well-known permissive histone modification, plays an important role in endotoxin tolerance by allowing upregulation of host beneficial genes in tolerized macrophages (11). Mixed-lineage leukemia 1 (MLL1) is a histone H3K4 methyl transferase, and WD repeat-containing protein 5 (WDR5) forms a core complex with MLL1 and is essential for catalyzing trimethylation of H3K4 on chromatin (53). We showed that EPO induced WDR5 expression in tolerized macrophages and it was blocked by AKT inhibitor MK2206. Consistent with our results, experiments in colorectal cancer showed that WDR5 expression could be increased through activating PI3K/AKT signaling (54). We further determined whether WDR5-induced methylation contributed to EPO-mediated functional re-programming of tolerant macrophages. We found that blocking WDR5 with WDR5-0103 or treatment with a demethylating agent (5-AZA) in tolerized macrophages remarkably dampened EPO-mediated functional re-programming, as indicated by diminished *Cnlp*, *Marco*, and *Vegfc* upon LPS restimulation, whereas proinflammatory *Il1b*, *Il6*, and *Tnfa* were not affected. Thus, we present a novel function of WDR5 in the contribution of EPO-mediated re-programming in tolerant macrophages.

The current study has some limitations. For instance, the effects of post-treatment should be validated scientifically, and the sample size should be increased for further investigations. In addition, increasing scientific literature has demonstrated that β common receptor (βCR) plays a crucial role in EPO-mediated protective effects by forming a heterodimeric receptor with EPOR (EPOR/βCR) (55). However, there is still controversy on how βCR interacts with EPOR. For example, a recent study demonstrated that the extracellular regions of the EPOR and the βCR do not specifically associate and that EPO does not promote interaction between the EPOR and the βCR (56). Therefore, further investigation would be required to confirm the possible involvement of EPOR/βCR in the mediation of macrophages endotoxin tolerance.

In summary, our present data indicate that EPO functionally re-programs endotoxin-tolerant macrophages through PI3K/AKT pathway–induced upregulation of *Irak3* and *Wdr5*. We report that EPO protected LPS-tolerized mice from secondary infection of *E. coli* and improved the outcomes of septic mice. Our findings open further research of this drug to new opportunities beyond the limit of its actual clinical utility. However, additional research would be needed to transfer our findings into clinical settings.

## Data availability statement

The raw data supporting the conclusions of this article will be made available by the authors, without undue reservation.

## Ethics statement

The animal study was reviewed and approved by Laboratory Animal Welfare and Ethics Committee of the Army Medical University.

## Author contributions

BL provided the idea and conceived and designed the experiments. XZ, DH and JJ performed the experiments. FL, JM, WL, TL, YL, and ZW provided the technical support. BL, ZZ and FZ analyzed and interpreted the data. BL wrote the draft of the manuscript. ZZ and FZ revised the manuscript. BL, FZ and ZZ supervised the study. All authors contributed to the article and approved the submitted version.

## Funding

This work was supported by grants (32000638 and 82071778) from the National Natural Science Foundation of China (BL and ZZ).

## Conflict of interest

The authors declare that the research was conducted in the absence of any commercial or financial relationships that could be construed as a potential conflict of interest.

## Publisher’s note

All claims expressed in this article are solely those of the authors and do not necessarily represent those of their affiliated organizations, or those of the publisher, the editors and the reviewers. Any product that may be evaluated in this article, or claim that may be made by its manufacturer, is not guaranteed or endorsed by the publisher.
