# Supplementary material for: Erythropoietin mediates re-programming of endotoxin-tolerant macrophages through PI3K/AKT signaling and protects mice against secondary infection
Source: Front Immunol. 2022 Aug 9;13:938944. doi: 10.3389/fimmu.2022.938944 (PMC9396350; doi:10.3389/fimmu.2022.938944)
Supplement: Supplementary file 1 [file DataSheet_1.docx]

**Supplementary Figure legends**

**
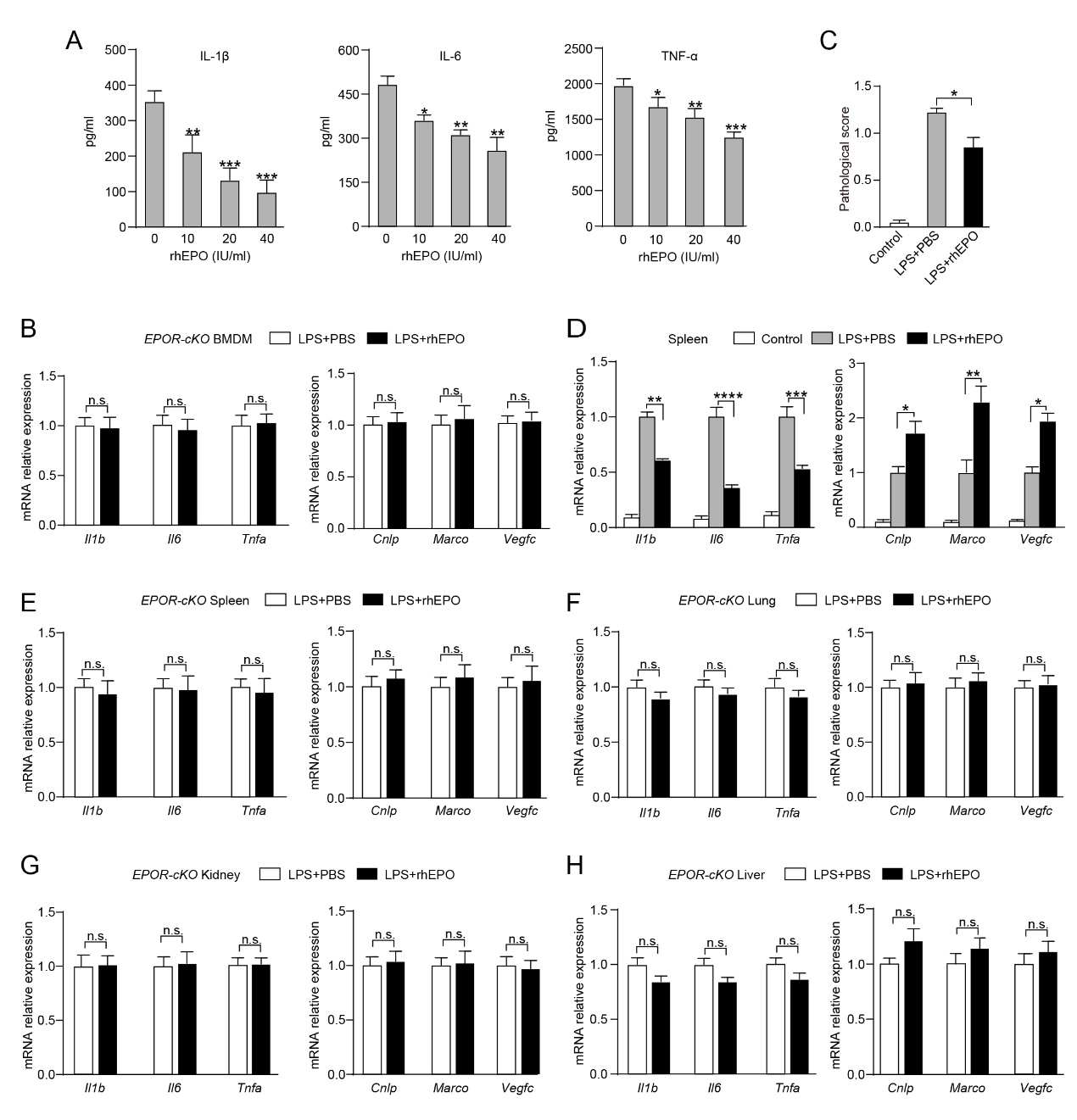
**

**Figure S1. EPO failed to promote endotoxin tolerance in *EPOR-cKO* mice*.***

**A**: *In vitro* cultured RAW264.7 macrophages were incubated with different dose of rhEPO (0, 10, 20 and 40 IU/ml) in presence of LPS (100 ng/ml) for 24 hours, then cells were washed with PBS for twice followed by a secondary LPS stimulation (10 ng/ml) for 6 hours, IL-1, IL-6 and TNF-α concentrations in culture medium were measured by ELISA (n = 3). **B**: *In vitro* cultured BMDMs from *EPOR-cKO* mice were incubated with rhEPO (40 IU/ml) in presence of LPS (100 ng/ml) for 24 hours, then cells were washed with PBS for twice followed by a secondary LPS stimulation (10 ng/ml) for 6 hours, gene expression was measured by qRT-PCR (n = 3). **C:** Histological evaluation for lung specimens stained with HE in Figure 2C. **D**: WT C57/BL6 mice were intraperitoneally injected with LPS (1 mg/kg) together with rhEPO (5000 IU/kg) or PBS for 24 hours, then these mice were intraperitoneally given with a secondary LPS injection (10 mg/kg) for 6 hours, gene expression in mice spleen was measured by qRT-PCR (n = 3). **E-H**: *EPOR-cKO* mice were intraperitoneally injected with LPS (1 mg/kg) together with rhEPO (5000 IU/kg) or PBS for 24 hours, then these mice were intraperitoneally given with a secondary LPS injection (10 mg/kg) for 6 hours, gene expression in mice spleen (E), lung (F), kidney (G) and liver (H) was measured by qRT-PCR (n = 3). Data are representative of three independent experiments. Results were expressed as mean ± SEM. *P < 0.05, **P < 0.01, ***P < 0.001 and ****P < 0.0001. n.s.: not statistically significant. Statistics: one-way (A, C) or two-way (B, D-H) ANOVA with Tukey’s post hoc test for multiple comparisons.

**
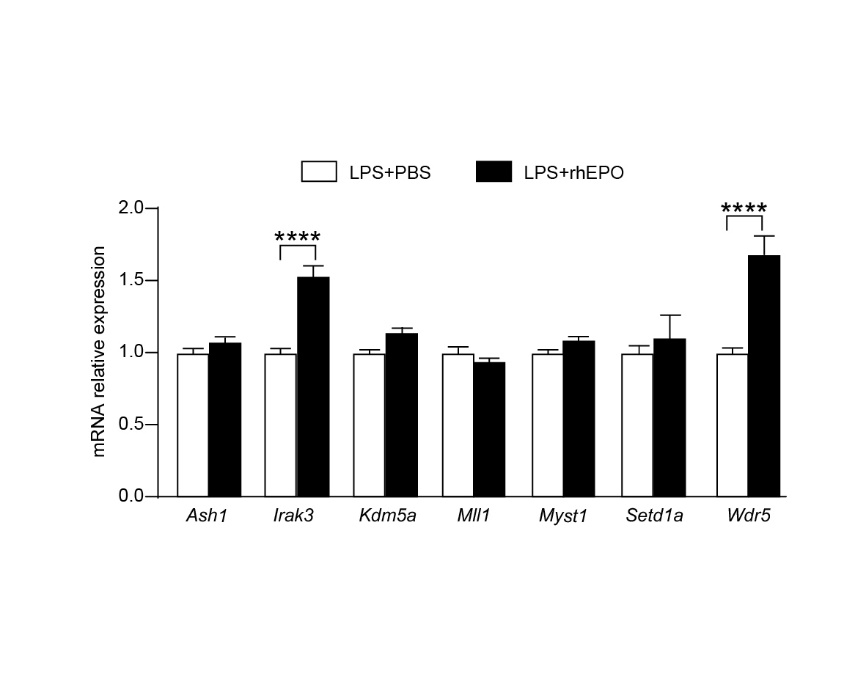
**

**Figure S2. EPO upregulated mRNA expression of *Irak3* and *Wdr5* in LPS-tolerized macrophages*.***

*In vitro* cultured RAW264.7 macrophages were incubated with rhEPO (40 IU/ml) or PBS in presence of LPS (100 ng/ml) for 24 hours, then gene expression was measured by qRT-PCR (n = 3). Results were expressed as mean ± SEM. *P < 0.05, **P < 0.01, ***P < 0.001 and ****P < 0.0001. Statistics: two-way ANOVA with Tukey’s post hoc test for multiple comparisons.

**
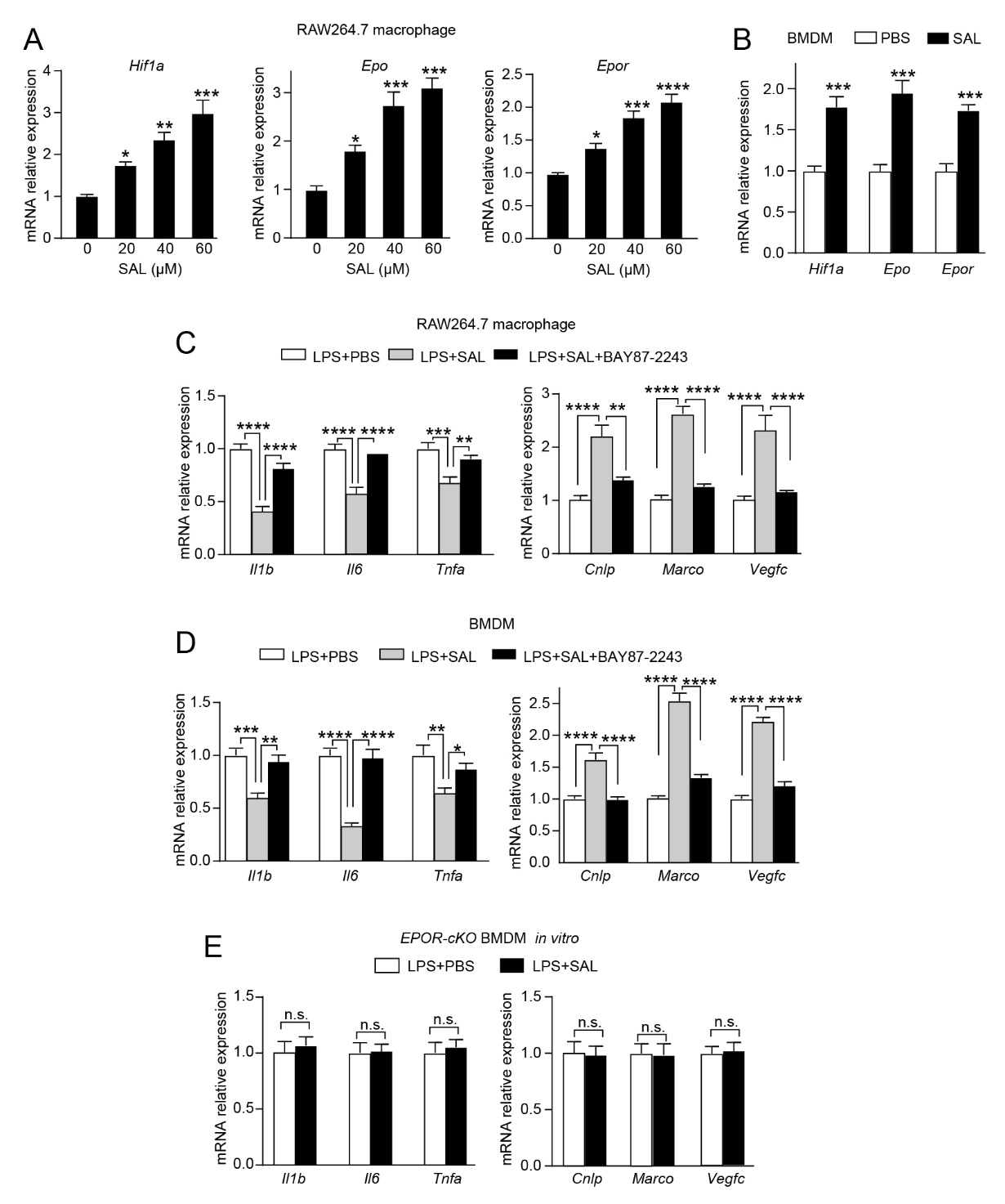
**

**Figure S3. SAL promoted endotoxin tolerance of macrophages through HIF-1α / EPO pathway.**

**A**: *In vitro* cultured RAW264.7 macrophages were incubated with SAL (0, 20, 40 and 60 μM) in presence of 100 ng/ml LPS for 24 hours, mRNA levels of *Hif1a*, *Epo* and *Epor* in macrophages were measured by qRT-PCR (n=3). **B**: *In vitro* cultured BMDMs from WT C57BL/6 mice were incubated with SAL (60 μM) in presence of 100 ng/ml LPS for 24 hours, mRNA levels of *Hif1a*, *Epo* and *Epor* were measured by qRT-PCR (n=3). **C-D**: *In vitro* cultured RAW264.7 macrophages (C) or BMDMs from WT C57BL/6 mice (D) were pretreated by 100 ng/ml LPS, together with SAL (60 μM), SAL (60 μM) + BAY87-2243 (20 μM) or PBS for 24 hours. Then cells were washed with PBS for twice followed by a secondary LPS stimulation (10 ng/ml) for 6 hours, and gene expression was measured by qRT-PCR (n=3). **E**: *In vitro* cultured BMDMs from *EPOR-cKO* mice were pretreated by 100 ng/ml LPS together with SAL (60 μM) or PBS for 24 hours. Then cells were washed with PBS for twice followed by a secondary LPS stimulation (10 ng/ml) for 6 hours, and gene expression was measured by qRT-PCR (n=3). Data are representative of three independent experiments. Results were expressed as mean ± SEM. *P < 0.05, **P < 0.01, ***P < 0.001 and ****P < 0.0001. n.s.: not statistically significant. Statistics: One-way ANOVA (A) or two-way ANOVA (B-E) with Tukey’s post hoc test for multiple comparisons.


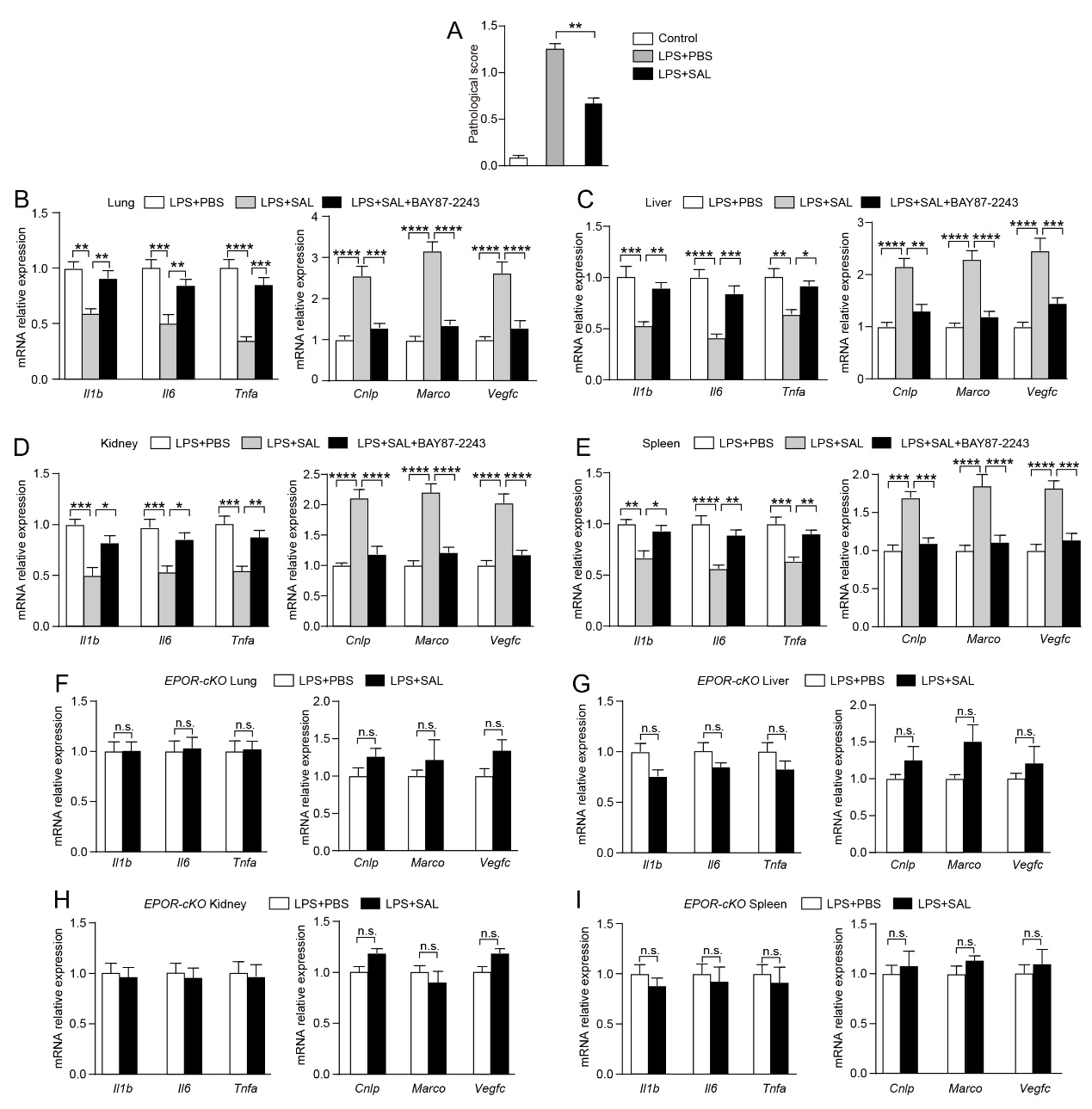


**Figure S4. SAL promoted mice endotoxin tolerance through HIF-1α / EPO pathway.**

**A:** Histological evaluation for lung specimens stained with HE in Figure 4C. **B-E**: WT C57BL/6 mice were intraperitoneally injected with LPS (1 mg/kg) + PBS, LPS (1 mg/kg) + SAL (40 mg/kg) or LPS (1 mg/kg) + SAL (10 mg/kg) + BAY87-2243 (9 mg/kg) for 24 hours, then these mice were intraperitoneally given with a secondary LPS injection (10 mg/kg) for 6 hours. qRT-PCR measurement of gene expression in mice lung (B), liver (C), kidney (D) and spleen (E). **F-I**: *EPOR-cKO* mice were intraperitoneally injected with LPS (1 mg/kg) together with SAL (40 mg/kg) or PBS for 24 hours, followed by a secondary LPS injection (10 mg/kg) for 6 hours, qRT-PCR measurement of gene expression in mice lung (F), liver (G), kidney (H) and spleen (I) (n = 3). Data are representative of three independent experiments. Results were expressed as mean ± SEM. *P < 0.05, **P < 0.01, ***P < 0.001 and ****P < 0.0001. n.s.: not statistically significant. Statistics: one-way (A) or two-way (B-I) ANOVA with Tukey’s post hoc test for multiple comparisons.
