# Supplementary material for: Erythropoietin mediates re-programming of endotoxin-tolerant macrophages through PI3K/AKT signaling and protects mice against secondary infection
Source: Front Immunol. 2022 Aug 9;13:938944. doi: 10.3389/fimmu.2022.938944 (PMC9396350; doi:10.3389/fimmu.2022.938944)
Supplement: Supplementary file 2 [file DataSheet_2.docx]

**Sequence of primers used.**

| Name | Sequence (Forward) | Sequence (Reverse) |
| --- | --- | --- |
| *Actb* | 5′-GGC TGT ATT CCC CTC CAT CG-3′ | 5′-CCA GTT GGT AAC AAT GCC ATG T-3′ |
| *Tnfa* | 5′-GAC GTG GAA GTG GCA GAA GAG-3′ | 5′-TGC CAC AAG CAG GAA TGA GA-3′ |
| *Hif1a* | 5′-GAA ATG GCC CAG TGA GAA AA-3′ | 5′-CTT CCA CGT TGC TGA CTT GA-3′ |
| *Il6* | 5′-TCT ATA CCA CTT CAC AAG TCG GA-3′ | 5′-GAA TTG CCA TTG CAC AAC TCT TT-3′ |
| *Il1b* | 5′-ATG GGC AAC CAC TTA CC-3′ | 5′-AAT GAA AGA CCT CAG TGC G-3′ |
| *Marco* | 5′-ACA GAG CCG ATT TTG ACC AAG-3′ | 5′-CAG CAG TGC AGT ACC TGC C-3′ |
| *Vegfc* | 5′-CAG TGT CAG GCA GCT AAC AAG-3′ | 5′-GGT CCA CAG ACA TCA TGG AA-3′ |
| *Cnlp* | 5′-CAC AGA CCC GCT GAG TTT TAT-3' | 5′-TGT CTA GGG ACT GCT GGT TGA-3′ |
| *Epor* | 5′-GGT GAG TCA CGA AAG TCA TGT-3′ | 5′-CGG CAC AAA ACT CGA TGT GTC-3′ |
| *Epo* | 5'-ATC TGC GAC AGT CGA GTT CT-3' | 5'-GTA TCC ACT GTG AGT GTT CG-3' |
| *Wdr5* | 5′-TTT GAA GAT TTG GGA CGT GAG TT-3'  5′-TTT GAA GAT TTG GGA CGT GAG TT-3' | 5′-ATG GGC AGG CAA AGT CTT GAG-3' |
| *Kdm5a* | 5′-CAC AGA CCC GCT GAG TTT TAT-3'  5′-CAC AGA CCC GCT GAG TTT TAT-3' | 5′-CTT CAC AGG CAA ATG GAG GTT-3' |
| *Irak3* | 5′-GAT GCT TTA TTC AAG TGA CCG C-3' | 5′-CTC TGT TCA CCT ATA CCA AGA TCT CA-3' |
| *Mll1* | 5′-GCA GAT TGT AAG ACG GCG AG-3' | 5′-GAG AGG GGG TGT TCC TTC CTT-3' |
| *Setd1a* | 5′-TGC TGT CCC TCG TAG ACT GG-3' | 5′-GGC TCT TTC CGT TTT ACC TTG A-3' |
| *Ash1* | 5′-CCT CGG TGG ACT AAA GTG GTG-3' | 5′-CGC TGG CTC AGA ACT ATT TGA-3' |
| *Myst1* | 5′-ACG AGG CGA TCA CCA AAG TG-3' | 5′-AAG CGG TAG CTC TTC TCG AAC-3' |

**ELISA**

The supernatant of cell culture medium was collected to detect the concentrations of IL-1β, IL-6 and TNF-α using ELISA kits (R&D Systems, Minneapolis, MN, USA) according to the manufacturer’s instructions.
